# Mechanisms and Therapeutic Targets of Hypoxia-Mediated Modifications in Glycolysis and Lactylation in Rheumatoid Arthritis

**DOI:** 10.3390/cells15121122

**Published:** 2026-06-22

**Authors:** Niqin Xiao, Heguo Yan, Yujiang Xi, Yundong Xu, Jian Zhang, Zhaofu Li, Zhaohu Xie

**Affiliations:** 1 Yunnan University of Chinese Medicine, Kunming 650500, China; xnq2022@126.com (N.X.);; 2 Joint Graduate School of Traditional Chinese Medicine of China, Suzhou 215105, China

**Keywords:** rheumatoid arthritis, hypoxia, glycolysis, lactylation, treatment strategies

## Abstract

Rheumatoid arthritis (RA) is an autoimmune disease primarily characterized by chronic, erosive polyarthritis. It is associated with a high rate of disability, and its pathogenesis remains incompletely understood. Uncontrolled chronic inflammation, synovial hyperplasia, Pannus formation, and bone destruction in RA patients remain the core challenges facing current clinical treatment, and the inflammatory response is generally considered the initiating factor for this series of pathological processes. In an inflammatory environment, the body’s metabolic rate accelerates, leading to increased local oxygen consumption and ultimately creating a hypoxic microenvironment. Research has shown that under hypoxic conditions, glycolysis serves as the body’s primary energy pathway and is essential for sustaining the inflammatory response. Furthermore, lactate, a byproduct of glycolysis, functions not only as a metabolic byproduct but also as a precursor molecule; through lactylation, it contributes to the progression of RA. Although this metabolic–epigenetic axis is a common feature of various chronic inflammatory diseases, its effects on joint pathology may contribute to RA progression. Therefore, this article focuses on the intrinsic connections among hypoxia, glycolysis, and lactylation, and systematically reviews the immunological and inflammatory mechanisms of glycolysis in RA, the relationship between glycolysis and synovial hyperplasia, Pannus formation, and bone destruction in RA, and the role of lactate modification in promoting the pathological progression of RA. It also summarizes the latest research advances in RA therapies targeting hypoxia, glycolysis, and lactate modification, aiming to provide a theoretical basis for a deeper understanding of the pathogenesis of RA and the development of targeted treatment strategies.

## 1. Preface

Rheumatoid arthritis (RA) is an autoimmune disease characterized by chronic, erosive polyarthritis [1,2,3]. If not actively treated, joint deformity and loss of function will eventually occur. The prevalence of RA worldwide is between 0.5% and 1% [4], and its prevalence is relatively higher in women and people with a family history of RA. The global incidence of RA is on the rise [5]. For patients with RA in China who have had the disease for more than 15 years, the disability rate reaches 61.3% [6], which imposes a substantial burden on patients’ physical and mental health as well as on family finances.

**Figure 1:**
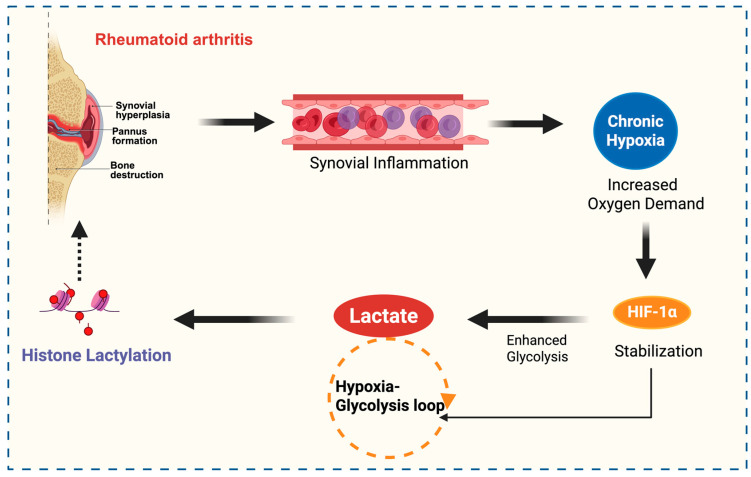
The role of hypoxia-mediated modifications in glycolysis and lactylation in RA.

RA is an immune-inflammatory disease characterized by immune dysregulation in genetically susceptible individuals triggered by environmental factors; its pathogenesis is complex. Current treatments cannot achieve a cure; instead, they rely on drug-based maintenance therapy to control symptoms and slow disease progression. Therefore, further exploration of RA-related pathogenic mechanisms and therapeutic targets remains a critical priority. The massive infiltration of immune cells into the synovium, synovial hyperplasia, and Pannus formation collectively lead to increased oxygen demand within the joint cavity, thereby causing persistent tissue hypoxia. Studies have reported that, compared to healthy individuals, the partial pressure of oxygen in the joint cavity of RA patients is significantly reduced [7]. Hypoxia, in turn, promotes the expression of glycolysis-related genes through key transcription factors such as hypoxia-inducible factor-1α (HIF-1α), creating a vicious cycle between hypoxia and glycolysis. While this metabolic process sustains the inflammatory response, its core end-product, lactate, accumulates locally in large quantities. Recent studies have revealed that lactate functions not only as a metabolic byproduct but also as a novel epigenetic modifier, mediating histone lactylation and participating in the pathological progression of RA [8]. Therefore, the metabolic–epigenetic axis—associated with hypoxia-induced glycolysis and lactylation—may play an important role in RA (Figure 1).

Consequently, this article focuses on the intrinsic connections among hypoxia, glycolysis, and lactylation, and systematically reviews the immunological and inflammatory mechanisms of glycolysis in RA, the relationship between glycolysis and synovial hyperplasia, Pannus formation, and bone destruction in RA, and the role of lactylation in promoting the pathological progression of RA. This review also summarizes the latest research advances in RA therapies targeting hypoxia, glycolysis, and lactylation, with the aim of providing a theoretical basis for a deeper understanding of the pathogenesis of RA and the development of targeted therapeutic strategies.

In RA, local activation of immune and inflammatory cells in the synovium leads to increased metabolic demands and oxygen consumption, resulting in local hypoxia. Under hypoxic conditions, HIF-1α is activated, promoting a shift toward enhanced glycolysis to sustain inflammatory responses. As a result, large amounts of lactate are produced during glycolysis. Beyond its metabolic role, lactate acts as a signaling molecule that mediates histone lactylation, thereby contributing to epigenetic regulation in RA. Lactylation may promote synovial inflammation, Pannus formation, and bone destruction, thereby exacerbating disease progression. These processes collectively form a positive feedback loop between hypoxia, glycolysis, and lactylation in RA.

## 2. The High Metabolic Rate Associated with Inflammation Leads to Hypoxia, Which, in Turn, Triggers Glycolysis

The core challenges in the current clinical management of RA patients remain uncontrolled chronic inflammation, synovial hyperplasia, Pannus formation and destruction of cartilage and bone. The inflammatory response is generally recognized as the initiating factor for this series of pathological processes. In RA, the synovium undergoes persistent hyperplasia due to chronic inflammation, characterized by abnormal proliferation of synovial cells and massive infiltration and accumulation of immune cells. The high metabolic activity of these cells dramatically increases the demand for oxygen [7], and as synovial hyperplasia progresses, the distance between old and new blood vessels increases, further elevating oxygen demand. Consequently, the local oxygen partial pressure in the joint decreases significantly [9]. Under hypoxic conditions, glycolysis becomes the primary energy supply pathway, providing crucial support for the maintenance of the inflammatory response [10,11]. Certain healthy lifestyle practices, such as aerobic exercises, including jogging and Tai Chi, can help improve patients’ overall condition [12]. However, their mechanisms of action remain incompletely understood. While the clinical benefits of these exercises are widely recognized as multifactorial, we conceptually hypothesize that they may also be related to enhancing systemic or local blood oxygen supply. If validated, such increased oxygen delivery could theoretically improve the local hypoxic microenvironment and inhibit glycolysis. Therefore, hypoxia may play a central role in the progression of RA, and modulating hypoxia could offer a new approach to the prevention and treatment of RA [13,14].

## 3. Glycolysis Is a Crucial Pathway for Initiating and Sustaining RA Inflammatory Responses

Normal cells generate energy through oxidative phosphorylation (OXPHOS) under aerobic conditions, with aerobic glucose oxidation serving as the primary pathway for energy production in normal synovial cells. Under anaerobic conditions, they switch to the glycolytic pathway [15]. In this condition, glucose undergoes a series of enzymatic reactions involving enzymes such as hexokinase II (HK2) and phosphofructokinase to convert into pyruvate and lactate, producing adenosine triphosphate (ATP). This metabolic process is termed anaerobic glycolysis. In rapidly proliferating abnormal cells within the human body—such as tumor cells, activated immune cells in RA, and synovial cells—energy is obtained through glycolysis rather than oxidative phosphorylation, even under aerobic conditions. This process, termed aerobic glycolysis or the Warburg effect, represents metabolic reprogramming [16,17,18].

Patients with RA often experience chronic inflammation, characterized by activated immune cells, increased metabolic demands, and elevated oxygen consumption, leading to a hypoxic microenvironment at the site of inflammation [19,20]. Under hypoxic conditions, the overall rate of glycolysis significantly increases to sustain the inflammatory response, concurrently activating HIF-1α [21,22,23]. During this metabolic shift, lactate dehydrogenase A (LDHA), acting as a key redox enzyme, catalyzes the reversible reduction of pyruvate to lactate [24,25,26,27]. This enzymatic reaction sustains high glycolytic flux and the associated production of ATP at the substrate level, thereby fueling the inflammatory response at the affected site. This process sustains or exacerbates inflammation, creating a vicious cycle. Pathological joint damage in RA patients, such as synovial hyperplasia, Pannus formation, and bone destruction, is closely linked to disease prognosis. Previous studies have demonstrated that glycolysis is associated with synovial hyperplasia, Pannus formation, and bone destruction in RA [28]. Consequently, glycolysis plays a vital role in RA, and downregulating its pathway activity substantially ameliorates disease severity [14,29]. Collectively, we propose that investigating RA pathogenesis through glycolysis regulation may provide novel therapeutic targets for RA prevention and treatment.

### 3.1. Glycolysis Is Involved in the Regulation of Immune Cell Activation in Inflamed Microenvironments

In RA, synovial and resident immune cells at the affected site abnormally secrete pro-inflammatory mediators, including inflammatory chemokines. Their primary energy supply shifts to glycolysis for rapid energy production. These metabolic alterations continuously recruit peripheral immune cells to the inflammatory site. The aggregated immune cells interact with each other, further destabilizing the inflammatory microenvironment. This is specifically manifested as follows: macrophages differentiated from monocytes polarize toward the pro-inflammatory M1 phenotype; neutrophil apoptosis is impaired; dendritic cells mature and secrete pro-inflammatory factors; B cells become hyperactivated to produce autoantibodies; CD4^+^ T cells exhibit imbalanced differentiation, skewing toward the Th17 subset, while Treg cell function is suppressed. These abnormal changes mutually reinforce each other, forming a self-perpetuating inflammatory cycle that ultimately contributes to persistent and exacerbated inflammation.

It has been found that glycolysis may be a potential mechanism for macrophage M1 polarization and inflammatory gene expression [30]. Typically, M1 macrophages utilize the glycolytic pathway to meet the high energy demands of their proinflammatory response. During RA progression, macrophages in inflamed joints appear to shift to a hypermetabolic glycolytic state as the M1/M2 ratio increases. The use of the glycolysis inhibitor 2-deoxy-D-glucose (2-DG) or knockdown of key glycolytic enzymes can inhibit the proinflammatory response of macrophages [31]. In neutrophils, hypoxic conditions inhibit neutrophil apoptosis by activating HIF-1α expression through the nuclear factors kappa B (NF-κB) signaling pathway [32]. The accumulation of apoptotic cells leads to the release of damage-associated molecular patterns (DAMPs), thereby overactivating the innate immune system, increasing inflammation, and exacerbating RA [33]. It is evident that dendritic cells (DCs) play a pivotal function in the development of RA [34]. Glycolysis can drive the transformation of normal dendritic cells into pro-inflammatory DCs, thereby inducing Th17 cell activation [35].

B cells participate in the pathogenesis of RA by producing autoantibodies, inducing T cells, secreting cytokines, and recruiting other inflammatory cells to tissues [36]. Some studies have found that activated B cells rapidly enhance glycolysis in response to antigenic stimulation to meet proliferation and antibody (Ab) synthesis requirements [37]. The induction of glycolysis is critical for Ab production because glycolytic inhibition of the pyruvate dehydrogenase kinase inhibitor dichloroacetic acid severely inhibits B cell proliferation and Ab secretion both in vitro and in vivo [38]. Research has found that B cells in mice with autoimmune serum-positive arthritis exhibit heightened glycolytic activity, and inhibiting glycolysis reduces the production of pathogenic autoantibodies [39].

**Figure 2:**
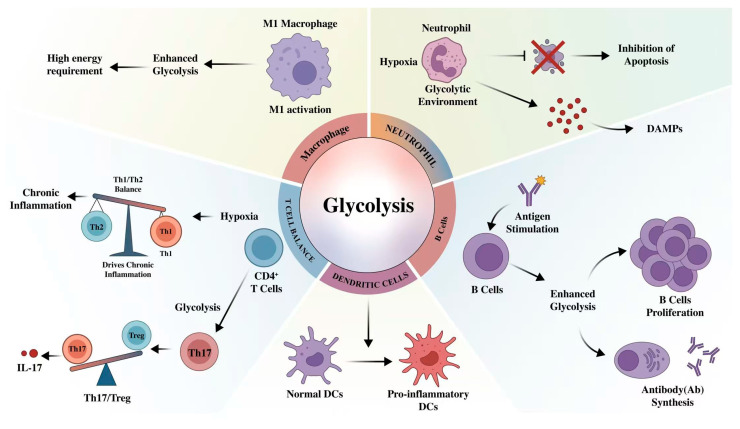
Glycolysis maintains the activation state of immune cells in RA.

Tissue hypoxia, as a core trigger of glycolysis, disrupts the balance between T helper cell 1 (Th1) and Th2 cells, leading to Th1-dominant effects that drive chronic inflammation [40]. Lactate produced by glycolysis accumulates in the synovium of RA joints, and lactate facilitates the conversion of CD4^+^ T cells into the IL-17 subpopulation [41]. In glycolysis, LDHA, as a key catalytic enzyme, promotes increased lactate production, which induces Th17 subset polarization [42], thereby generating excessive IL-17. HIF-1α controls metabolic checkpoints and induces glycolysis, a metabolic event that favors Th17 cell development but impedes Treg cell differentiation [43]. The Th17 subpopulation of CD4^+^ T cells is a key player in the mediation of chronic inflammation within tissues and organs [43,44,45]. Treg cells maintain immune tolerance and prevent excessive activation of the immune system [46] (Figure 2).

M1 macrophages rely on glycolysis to meet the high energy demands of their pro-inflammatory responses. Hypoxia and glycolytic conditions inhibit neutrophil apoptosis and promote the production of DAMPs. DCs transform into pro-inflammatory DCs when exposed to enhanced glycolysis. Upon antigen stimulation, activated B cells rapidly enhance glycolysis to meet the energy demands of proliferation and antibody (Ab) synthesis. Tissue hypoxia, as a key driver of glycolysis, can disrupt the balance between T helper cell 1 (Th1) and Th2 cells, leading to a predominance of Th1 effects that drive chronic inflammation. Glycolysis promotes the differentiation of CD4^+^ T cells into Th17 cells, causing an imbalance in the Th17/Treg ratio and thereby increasing IL-17 levels.

### 3.2. Glycolysis-Mediated Signaling Pathways Associated with Inflammatory Responses

**Figure 3:**
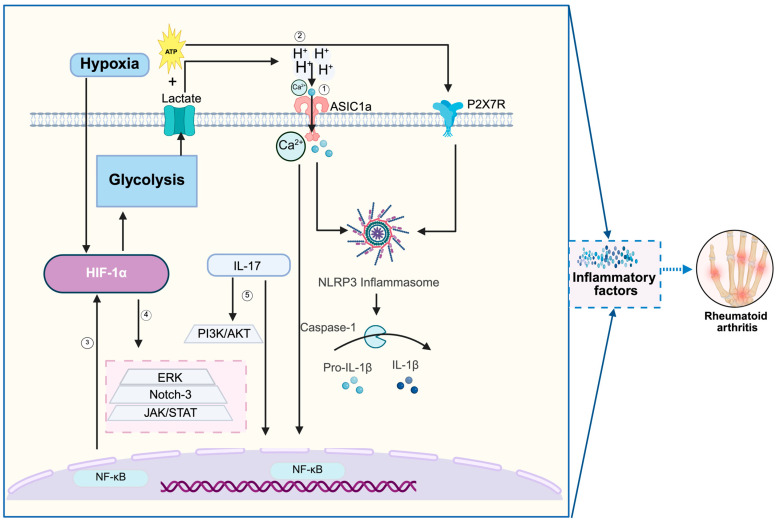
The process by which glycolysis initiates and maintains inflammatory responses.

Once glycolysis is activated, it leads to increased lactate release, thereby creating an acidic environment. An acidic environment can activate Acid-Sensing Ion Channel 1a (ASIC1a) and accelerate extracellular Ca^2+^ influx, leading to intracellular Ca^2+^ overload. This process fosters nuclear transcription of NF-κB and activates the NOD-, LRR- and Pyrin Domain-Containing Protein 3 (NLRP3) inflammasome. The outcome is the synthesis and secretion of pro-inflammatory cytokines, such as interleukin-1β (IL-1β), IL-6, and TNF-α, thereby inducing or exacerbating joint inflammation [47]. HIF-1α mRNA levels can be upregulated in an NF-κB-dependent manner [48,49]. Stimulation of immune cells with lipopolysaccharide can upregulate HIF-1α mRNA via NF-κB-dependent upregulation of HIF-1α to activate HIF-1α. IL-17, released by Th17 cells, is known to be activated through NF-κB and phosphatidylinositol 3-kinase/protein kinase B (PI3K/AKT) as well as other pathways, further promoting the secretion of IL-6 and IL-8 [50], and contributing to RA progression [51]. TNF-α contributes to leukocyte aggregation, induces secretion of IL-1β, IL-6, and synergistically enhances the inflammatory response [52]; IL-6, a key proinflammatory cytokine, is primarily produced by monocyte macrophages and enhances the inflammatory effects of IL-1 and TNF-α, thereby worsening RA inflammation. Pyruvate produced during glycolysis is converted to lactate by LDHA, accompanied by ATP production. The P2X7 receptor, an ATP-gated ion channel, plays a significant role in the inflammatory response by recruiting the NLRP3–cysteine-dependent aspartate-specific protease-1 (caspase-1) inflammasome complex [53]. Moreover, HIF-1α can maintain or exacerbate inflammation in the affected area by overproducing cytokines, such as TNF-α, IL-1, and IL-33, through pathways like extracellular signal-regulated kinase (ERK) and Notch homolog 3 (Notch-3) [54]. Research indicates that upregulating HIF-1α expression activates the Janus kinase (JAK)/Signal Transducer and Activator of Transcription (STAT) pathway [55]. JAK/STAT is a key pathway in RA pathogenesis [56,57], and studies have demonstrated that inhibiting this pathway suppresses crucial genes involved in glycolysis [58] (Figure 3).

① The increased production of lactate resulting from glycolysis creates an acidic environment, which, in turn, activates ASIC1a, accelerates the influx of extracellular Ca^2+^, and leads to intracellular Ca^2+^ overload. This process promotes the nuclear translocation and transcription of NF-κB, as well as the activation of NLRP3, thereby triggering the massive synthesis and secretion of inflammatory cytokines. ② Lactate and ATP are produced during glycolysis. The P2X7 receptor is a key ion channel that is triggered by ATP and is involved in the inflammatory response, mainly through the recruitment of the NLRP3–Caspase-1 complex. ③ HIF-1α mRNA levels can increase in an NF-κB-dependent manner. ④ HIF-1α can promote the excessive production and secretion of inflammatory cytokines through activation of the ERK signaling pathway, Notch-3 signaling pathway, and the JAK/STAT pathway, thereby maintaining or exacerbating inflammatory responses in the affected area. ⑤ IL-17 secreted by Th17 promotes the secretion of inflammatory cells through cellular pathways such as NF-κB and PI3K/AKT.

## 4. Glycolysis Is a Key Pathway Promoting Disease Progression

### 4.1. Synovial Hyperplasia and Pannus Formation

Persistent RA inflammation promotes the aggregation of leukocytes and the proliferation of synovial cells. Ciurtun et al. [59] reported a decrease in glucose levels and a significant rise in lactate content in RA synovial fluid, suggesting that the acidic microenvironment might lead to abnormal differentiation and proliferation of synovial tissue cells in RA [60]. Fibroblast-like synoviocytes (FLSs) play a crucial role in both synovial inflammation and joint damage [61], with prolonged inflammatory infiltration forming the pathological basis of persistent synovial inflammation. A study showed that blocking the NF-κB signaling pathway in collagen-induced arthritis (CIA) mice could downregulate NLRP3 and caspase-1, thereby reducing cellular pyroptosis and improving synovial inflammation [62]. Since glycolysis in RA patients can activate NLRP3 and Caspase-1, attenuating glycolysis might improve synovial inflammation. Liu et al. [63] found that an increase in HIF-1α was associated with a significant increase in Transforming Growth Factor-beta 1 (TGF-β1). Therefore, it is hypothesized that the elevation in HIF-1α levels resulting from excessive glycolysis activation in RA patients might lead to the overexpression of TGF-β1, an important mediator of synovial hyperplasia, thus exacerbating synovial hyperplasia.

**Figure 4:**
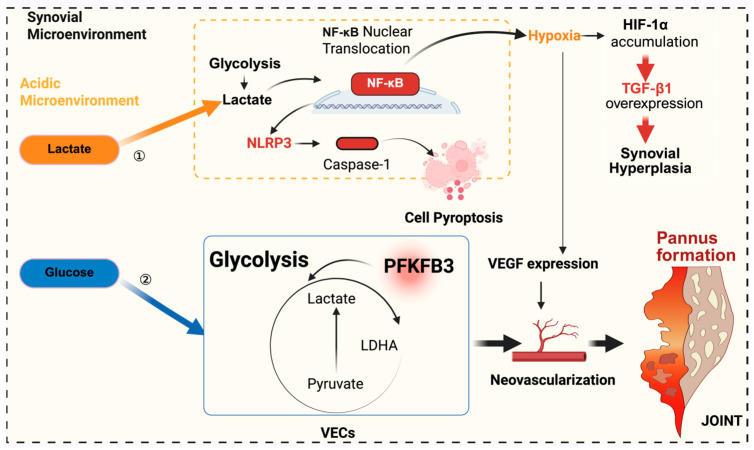
Glycolysis promotes synovial hyperplasia and Pannus formation.

Glycolysis, a form of metabolic reprogramming, is closely linked to neovascularization [64,65]. 6-phosphofructo-2-kinase/fructose-2,6-bisphosphatase 3 (PFKFB3) is a pivotal enzyme in glycolysis-promoting neovascularization. Moreover, pyruvate, a glycolysis product, also stimulates neovascularization. Lactate, produced through glycolysis, further promotes neoangiogenesis [66,67]. Vascular Endothelial Cells (VECs) primarily rely on glycolytic metabolism due to their lower mitochondrial content compared to other cells [68], allowing glycolysis to generate ATP more rapidly than OXPHOS. This metabolic choice is also an adaptation of VECs to inflammation. Studies have shown that under hypoxic conditions, the expression level of vascular endothelial growth factor (VEGF) increased in rabbits and rats [69], promoting the migration, proliferation, and angiogenesis of VECs. Glycolysis-induced neoangiogenesis leads to Pannus formation, and the attachment of Pannus to the articular cartilage surface can cause irreversible damage [70,71] (Figure 4).

① Lactate promotes the nuclear translocation of NF-κB, activates the NLRP3 inflammasome, and triggers pyroptosis via Caspase-1. Simultaneously, by inducing hypoxia, it activates HIF-1α, leading to increased TGF-β1 levels, which ultimately results in synovial hyperplasia. ② The key glycolytic enzyme PFKFB3, as well as its metabolic products pyruvate and lactate, can promote angiogenesis; among these, LDHA plays a pivotal role by catalyzing the reduction of pyruvate to lactate, thereby driving extracellular acidification. VECs rely more heavily on glycolysis for rapid energy supply to adapt to the inflammatory microenvironment. Under hypoxic conditions, increased VEGF expression enhances angiogenesis; glycolysis-driven abnormal angiogenesis leads to an increase in pannus formation.

### 4.2. Glycolysis Affects Bone Metabolism and Promotes Bone Destruction

In RA pathogenesis, bone destruction signifies disease severity and ultimately results in joint deformities and limited functional mobility, thus increasing the disability rate. Recent research on RA-related bone destruction has been refined, focusing primarily on cartilage, subchondral bone, and bone tissue. Articular cartilage is essential for maintaining joint motor function, and provides low-resistance, lubricated surfaces that facilitate smooth sliding, rolling, and stress transfer between the articular surfaces, thereby acting as an important cushion [72]. The subchondral bone is located adjacent to the articular cartilage, with the calcified cartilage zone acting as a transition between these two structures [73]. When the stress load becomes excessive, the subchondral bone can help prevent damage to the articular cartilage to some extent. Bone tissue, characterized by its dense texture and greater pressure resistance, is the main component of bone. Consequently, cartilage, subchondral bone, and bone tissue play key roles in the bone destruction process in RA patients.

#### 4.2.1. Cartilage Destruction

The excessive formation of Pannus leads to its invasion of intra-articular cartilage, resulting in cartilage destruction. Under hypoxic conditions, synoviocytes secrete large amounts of matrix metalloproteinases (MMPs), including MMP-1, MMP-2, MMP-3, MMP-9, MMP-12, and MMP-13, which degrade articular cartilage. Both HIF-1α and HIF-2α were found to be increased in the synovial cells of RA patients, as revealed by experimental and clinical data [74]. HIF-2α significantly enhances the production of various MMPs and polyprotein polyglucosidase-1 in synoviocytes [75,76]. MMP-13 specifically cleaves a range of extracellular matrix (ECM) proteins, leading to ECM remodeling [77]. The ECM, a key component of the cellular microenvironment, stores signaling molecules that can release and activate various inflammatory mediators when hydrolyzed, contributing to tissue damage [78,79]. It has been shown that excessive ECM degradation is observed in osteoarthritis [80] and that ECM degradation is a key pathologic feature of cartilage degeneration in OA. HIF-2α enhances Fas expression, promoting chondrocyte apoptosis and regulating autophagy in mature chondrocytes [81]. It is worth noting that while HIF-1α orchestrates the glycolytic phenotype of synovial cells, HIF-2α specifically triggers the catabolic cascade in the joint cartilage.

Glycolysis supports the differentiation of Th17 cells, and Th17 cells further promote RA osteoclast formation and cartilage erosion [82]. Studies have shown [10,39,41,83,84] that glycolysis is more active in the joint cavity of patients with RA, inhibiting apoptosis and maintaining the differentiation and activity of inflammatory cells by targeting protein 53 (p53). This increases the proportion of cells like T follicular helper cells (Tfh) and Th17, thereby exacerbating synovial inflammation in RA. Furthermore, elevated serum lactate levels in RA patients can contribute to an acidic microenvironment, a major consequence of glycolysis. The acidic microenvironment can further activate the expression levels of the anti-apoptotic gene Bcl-2 family through the acid-sensitive receptor GPR65, which inhibits apoptosis, resulting in the continuous proliferation of inflammatory cells [85]. Studies have shown that the presence of abundant inflammatory factors in the synovial tissue of RA patients contributes to the destruction of cartilage [86].

#### 4.2.2. Subchondral Bone Degeneration

In recent years, MRI studies of the joints have revealed that the subchondral bone represents a significant site of inflammation in RA, and bone marrow edema of the subchondral bone is closely associated with articular bone destruction [87,88,89]. Bailey et al. [90] discovered that activated TGF-β could affect the homeostasis of the subchondral bone plate through osteoblasts. Similarly, Dai et al. [91] found that TGF-β signaling was activated in the subchondral bone under conditions of partially or completely defective cartilage. Research has revealed that TGF-β activity is induced under hypoxic conditions [92], and increased HIF-1α expression promotes TGF-β expression [74]. RA synovial fluid contains elevated levels of TGF-β1, which has the capacity to activate the Smad2/3 signaling pathway in RA-FLS, thereby influencing bone remodeling [93]. Therefore, the hypoxic environment associated with RA inflammation promotes subchondral bone calcification, reducing its protective cushioning function.

#### 4.2.3. Bone Tissue Destruction

**Figure 5:**
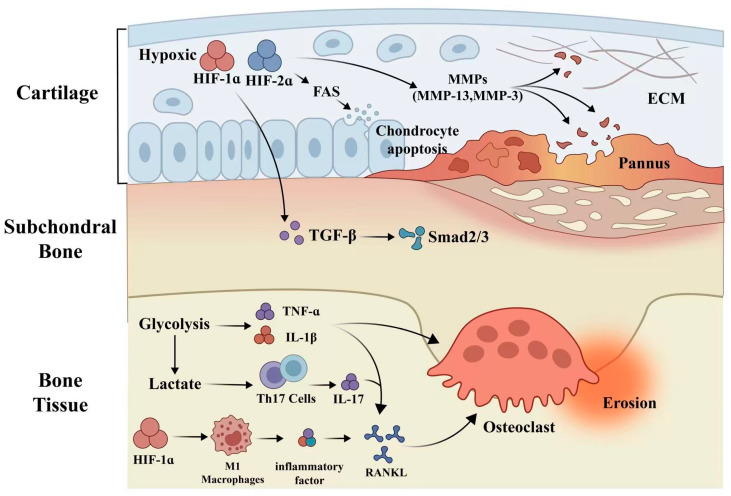
Glycolysis promotes bone destruction.

Osteoclasts play a crucial role in bone destruction [94]. Hypoxia in RA induces glycolysis, producing large amounts of inflammatory factors, like TNF-α and IL-1β. The acidic environment promotes the nuclear translocation and activation of NF-κB, while lactate drives the conversion of CD4^+^ T cells to the IL-17 subpopulation. Local inflammatory cytokines can increase osteoclast numbers [95]. The NF-κB signaling pathway directly induces osteoclast precursors to form osteoclasts and indirectly stimulates osteoclastogenesis by inducing stromal cells, osteoblasts, and activated T cells to express macrophage colony-stimulating factor and Receptor Activator of Nuclear Factor-κB Ligand (RANKL) [96]. RA patients experience a significant increase in IL-1β, a potent osteoclastogenic cytokine that directly or indirectly promotes osteoclast differentiation by upregulating the expression levels of RANKL in stromal cells [97]. Bone destruction is modulated when osteoprotegerin, a decoy receptor for RANKL, is administered to a rat model of arthritis [98]. Th17 cells produce IL-17, which mediates the expression of pro-inflammatory cytokines in innate immune cells, creating an inflammatory microenvironment that promotes local osteoclast differentiation. This process activates osteoclasts by inducing RANKL expression in synovial fibroblasts, driving bone destruction [99]. Bone homeostasis in RA is disrupted, mainly as a result of an imbalance between osteoblasts and osteoclasts [100]. Hypoxia in RA also promotes M1 macrophage polarization by activating HIF-1α [101], with M1-type cells secreting pro-inflammatory factors that regulate bone resorption. This induces RANKL expression and further stimulates osteoclast differentiation, resulting in abnormal bone metabolism in vivo and accelerated bone erosion and destruction [102]. Zha et al. [103] found that TNF-α could activate osteoclasts and accelerate the process of osteoporosis. Studies indicated a global osteoporosis prevalence of approximately 27.6% in RA [104], increasing fracture risk. Therefore, immune dysfunction in RA patients, along with synovial hyperplasia, Pannus formation, cartilage, subchondral bone, and bone tissue destruction, exacerbates abnormal bone metabolism and osteoporosis (Figure 5).

Excessive proliferation of vascularized tissue invades articular cartilage; under hypoxic conditions, synovial cells upregulate the expression of HIF-1α and HIF-2α. HIF-2α promotes the production of various MMP-13 and MMP-3, leading to degradation and remodeling of the ECM, the release of inflammatory mediators, and the exacerbation of cartilage damage. Concurrently, HIF-2α enhances Fas expression to mediate chondrocyte apoptosis. In hypoxic conditions, the subchondral bone region undergoes calcification and structural abnormalities via the HIF-1α/TGF-β/Smad2/3 signaling pathway. On the other hand, hypoxia in RA induces glycolysis, leading to the production of large amounts of inflammatory factors such as TNF-α and IL-1β. TNF-α and IL-1β directly or indirectly promote osteoclast differentiation and activation by upregulating RANKL expression; Lactate promotes Th17 cell differentiation, and IL-17 production is subsequently enhanced, which further upregulates RANKL expression to activate osteoclasts, thereby contributing to bone destruction. HIF-1α promotes M1 polarization of macrophages. M1 macrophages secrete various pro-inflammatory factors that induce RANKL expression. Upregulated RANKL expression further stimulates osteoclast differentiation, ultimately leading to bone destruction.

## 5. The Role of Lactylation in the Pathological Process of RA

Lactate not only plays a role in maintaining the homeostasis of cellular energy metabolism but also participates in various immune and inflammatory processes, serving as a key immunometabolic mediator in the inflammatory microenvironment [105]. During metabolic processes, lactate can also induce a new epigenetic modification—lactylation [106].

Histone lactylation is regulated by a dynamic enzymatic system linking cellular metabolism to epigenetic regulation. The histone acetyltransferase p300 has been identified as a key “writer” enzyme responsible for catalyzing lysine lactylation under high lactate conditions, thereby directly connecting glycolytic metabolism to transcriptional activation [107]. In contrast, histone deacetylases (HDACs) and members of the sirtuin family, particularly Sirtuin 3 (SIRT3), have been proposed as potential “eraser” enzymes that may reverse lactylation-associated transcriptional programs, although their precise catalytic roles in delactylation remain under active investigation [107,108]. This modification alters the epigenetic structure of histones without changing the gene sequence and has been shown to directly regulate gene expression [109]. Since histone lactylation is influenced by lactate levels and lactate production depends on glycolysis, this modification is regarded as a bridge between metabolism and gene transcription [110].

Compelling experimental evidence has recently substantiated the hyper-activation of this pathway in both clinical scenarios and animal models [8]. In clinical settings, comparative analyses of synovial tissue biopsies from RA patients demonstrated a profound upregulation of global histone lactylation, with specific enrichment of Histone H3 lysine 9 lactylation (H3K9la) in primary FLS compared to healthy controls [8]. Parallelly, in preclinical experimental animal models of RA, such as the CIA mouse model, evaluation of joint tissues revealed an accumulation of lactylated histones within the joint synovium [8]. This experimental increase in histone lactylation closely corresponds to the peak phase of inflammatory cell infiltration, joint swelling, and cartilage degradation, demonstrating its active participation in vivo [8].

Studies have shown that lactylation plays a significant immunoregulatory role in the inflammatory microenvironment. HMGB1, as an important DAMP, is significantly elevated in RA synovial fluid and acts as a key pro-inflammatory mediator [111]. Lactylation of HMGB1 leads to an increase in Neutrophil Extracellular Traps (NETs), thereby enhancing the inflammatory response [112]. Lactylation of NFATc2 promotes the migration of RA-FLS [8]. Furthermore, lactylation is also involved in the angiogenesis process. Studies have shown that H3K9la in endothelial cells is crucial for VEGF-induced angiogenesis [67]. In RA synovium, abnormal angiogenesis is a key basis for the formation of vascular plexuses; therefore, lactylation may play a critical role in this process. On the other hand, the lactylation of NFATc2 exacerbates the progression of RA by enhancing the invasive capacity of FLS toward cartilage [8]. In summary, lactylation may participate in the pathological process of RA by regulating multiple pathways, including inflammatory responses, synovial hyperplasia, angiogenesis, and bone destruction, suggesting that it may be a potential therapeutic target for RA.

Despite the growing body of evidence supporting the involvement of histone lactylation in the pathogenesis of RA, several important limitations should be acknowledged. First, most current studies demonstrate a strong association between increased lactylation levels and inflammatory activity; however, direct evidence establishing a causal role remains limited. Second, a substantial proportion of the available data is derived from in vitro experiments or animal models, while direct clinical validation in RA patients remains insufficient.

## 6. Therapeutic Strategies for RA Targeting Hypoxia, Glycolysis, and Lactate Modulation

### 6.1. Improving the Hypoxic Microenvironment

#### 6.1.1. Pharmacological Treatment to Improve Hypoxia

Current research indicates that certain natural medicines and their active components show potential for improving the hypoxic microenvironment in RA. Curcumin, a natural polyphenolic compound, not only possesses anti-inflammatory and antioxidant properties but also inhibits the activation of the NF-κB signaling pathway. This reduces the expression of HIF-1α and its downstream target VEGF, thereby decreasing angiogenesis and inflammatory responses and inducing FLS apoptosis, which helps alleviate pathological damage caused by synovial hypoxia to some extent [113,114]. Rhodiola and its active component, rhodiolin, possess anti-hypoxic, antioxidant, and immunomodulatory effects. Mechanistically, rhodiolin exerts its anti-hypoxic effects through multiple pathways. It can modulate the HIF-1α signaling pathway by reducing HIF-1α stability under hypoxic conditions [115]. Furthermore, rhodiolin modulates the PI3K/Akt signaling pathway and alleviates oxidative stress by reducing the accumulation of inflammatory factors, which further enhances cellular adaptation to hypoxia. Functionally, these effects are reflected in the inhibition of FLS proliferation and migration, reduced secretion of pro-inflammatory cytokines, and alleviation of synovial inflammation and metabolic stress in RA [116]. Therefore, rhodiola may exert a protective effect by improving the local hypoxic microenvironment.

#### 6.1.2. Physical Therapy and Exercise Therapy to Improve Hypoxia

Physical therapy is also an important adjunctive approach for improving the hypoxic microenvironment in RA. Studies have shown that hyperbaric oxygen therapy can increase local oxygen levels to reduce angiogenesis, primarily by accelerating the degradation of HIF-1α and inhibiting the production of VEGF-A [117]. However, the benefits of hyperbaric oxygen therapy are not permanent [118]; therefore, it is crucial to use other physical modalities to improve local hypoxia. Moxibustion has demonstrated potential for modulating the hypoxia–angiogenesis axis. Studies indicate that moxibustion can reduce serum HIF-1α and VEGF levels in RA patients [119]. Therefore, we hypothesize that thermal therapies such as moxibustion and local moist heat applications may alleviate pain and morning stiffness and promote local circulation, potentially by enhancing tissue perfusion and ameliorating synovial hypoxia.

Exercise therapy is also an important component of non-pharmacological management for RA. The 2022 ACR guidelines strongly recommend sustained, regular exercise for RA patients and conditionally recommend aerobic exercise, resistance training, and aquatic exercise [120]; certain healthy lifestyle practices, such as jogging, tai chi, massage, and physical therapy, offer benefits for improving patients’ overall condition [12]. These exercise therapies may indirectly alleviate hypoxia-related pathological processes by improving systemic oxygen utilization and the microcirculatory environment.

### 6.2. Potential Therapeutic Strategies Targeting Glycolysis

HK2 is a key enzyme in glycolysis, and inhibition of HK2 reduces glycolytic flux, thereby inhibiting pro-inflammatory immune cell activation. A glucose analogue known as 2-Deoxy-D-glucose (2-DG) competitively inhibits HK2. 2-DG has completed an early clinical trial evaluating its potential in cancer and inflammatory diseases [121]. At the same time, experimental studies have found that preventive inhibition of 2-DG significantly reduces joint inflammation [39]. HIF-1α is a key transcription factor regulating glycolysis; its inhibition suppresses glycolytic activity. PX-478 is a HIF-1α inhibitor, which was introduced as the first clinical-stage novel HIF-1α inhibitor for the treatment of solid tumors [122]. Cancer cells utilize the HIF-1α pathway to drive cancer progression through various mechanisms, such as promoting angiogenesis, metabolic reprogramming, and immune evasion. Therefore, it can provide a rationale for targeting HIF-1α in RA. Several previous studies have shown that fasting blood glucose and insulin levels improved in RA patients after treatment with TNF-α inhibitors [123,124]. This may be attributed to TNF-α inhibitors indirectly modulating glycolytic metabolism, which is closely associated with inflammation, by reducing inflammatory responses..

Methotrexate (MTX), a first-line disease-modifying anti-rheumatic drug (DMARD) agent, significantly downregulates the expression of HK2 and glucose and fructose carriers in the FLS of RA, thereby inhibiting glycolysis [125]. Tofacitinib, as a JAK/STAT pathway inhibitor, is widely used in clinical practice. Research has found that this drug can inhibit key glycolytic genes such as HK2 [58]. These data support the relationship between inflammation and abnormal glucose metabolism.

### 6.3. Inhibiting Lactate-Mediated Signaling for the Treatment of RA

Gastrodin targets Lysine Acetyltransferase 8 (KAT8) to inhibit H3K9la, thereby alleviating RA [126]. Beta-sitosterol inhibits the proliferation and migration of synovial cells in RA by lactating Glycosylphosphatidylinositol (GPI) [127]. Geniposidic acid (GPA) inhibits glycolysis, leading to reduced lactate production and decreased lactylation; this inhibitory effect suppresses M1 polarization of macrophages [128]. SIRT3 inhibits the activation of FLS in RA by suppressing H3K18la [129]. Parishin E (PE), extracted from Gastrodia elata, exerts a mitigating effect on RA by inhibiting macrophage polarization through the regulation of cellular glycolysis and the suppression of lactate modification at histone H3 sites (particularly H3K18 and H3K27) [130]. Luteolin-mediated inhibition of LDHA activity disrupts glycolysis-driven H3K9la, thereby silencing NFATC2, which, in turn, inhibits Th17 cell differentiation and central nervous system infiltration to alleviate RA-associated chronic pain [131].

### 6.4. Translational Barriers and Limitations of Metabolic Targeting

Transforming preclinical targets within the hypoxia–glycolysis–lactylation axis into viable human therapies presents several critical challenges. The foremost obstacle is the lack of systemic selectivity. Systemic administration of direct glycolytic inhibitors (2-DG) or potent HIF-1α blockers (PX-478) poses severe risks of off-target toxicities and impaired tissue repair.

Furthermore, the level of evidence supporting these therapeutic strategies varies significantly. While conventional DMARDs (methotrexate) and JAK inhibitors (tofacitinib) boast well-established clinical profiles, their metabolic effects are largely indirect. Conversely, novel small molecules and natural extracts that directly modulate enzymatic pathways or histone lactylation remain restricted to in vitro or rodent models. Overcoming these limitations will fundamentally depend on the innovation of advanced, joint-targeted drug delivery platforms.

## 7. Discussion and Summary

RA is an immune-mediated inflammatory disease resulting from the combined effects of genetic susceptibility and environmental factors. The inflammatory environment within the joint cavities of RA patients increases local oxygen consumption, leading to hypoxia at the affected sites. As the primary energy source in hypoxic conditions, glycolysis has been shown in a growing body of research to be involved in many important pathophysiological processes of RA, and its regulatory mechanisms are closely linked to the pathogenesis of the disease. Glycolysis not only provides energy for synovial cells and immune cells in RA but also contributes to disease progression through various mechanisms, including promoting the activation of inflammatory cells, synovial hyperplasia, Pannus formation, and accelerated bone destruction. Key glycolytic enzymes such as HK2 and PFKFB3 are upregulated in RA, and their regulatory networks are closely linked to inflammatory signaling pathways, suggesting they may serve as potential therapeutic targets. Furthermore, the accumulation of lactate, a byproduct of glycolysis, not only alters the local microenvironment but also participates in epigenetic regulation by inducing protein lactylation. As a novel post-translational modification linking metabolism to gene expression, lactylation can modulate the expression of inflammation-related genes and the pathological progression of RA, playing a significant role in the pathogenesis of RA.

Emerging evidence indicates that extracellular lactate acts as an anti-inflammatory negative feedback signal that stabilizes Treg cells, while baseline HIF-1α activation remains indispensable for chondrocyte survival and matrix synthesis within the avascular joint zone [132,133]. This biological duality highlights a fundamental conundrum in RA immunometabolism. Crucially, although a robust association links tissue lactate accrual and concomitant lactylation to RA progression, establishing definitive causality remains a formidable pharmacological challenge. Resolving this bottleneck is critical; future therapeutic strategies must pivot away from broad metabolic suppression toward precision interventions capable of selectively dissecting pathogenic circuits without dismantling these innate, protective feedback networks, thereby elucidating the exact underlying causal hierarchy.

Increased physical activity in patients with morning stiffness can improve blood oxygenation and circulation; oxygenating the body may alleviate pain and potentially offer new therapeutic insights for RA. Therefore, it is hypothesized that hypoxia-induced glycolysis and lactylation may contribute to the persistence and progression of arthritis in RA patients. Consequently, the regulation of glycolysis and lactylation modifications under hypoxic conditions offer new research avenues for elucidating the etiology, diagnosis, and treatment of RA. More importantly, they may serve as potential therapeutic targets for refractory RA, alleviating the symptoms of persistent or worsening arthritis in patients with refractory RA.

In conclusion, the hypoxia–glycolysis–lactylation axis offers a highly dynamic framework for understanding RA chronicity. To further expand the future clinical translational value of this topic, it is imperative to integrate this metabolic–epigenetic axis with recent high-resolution omics advancements. Specifically, while global immunometabolism dictates the overall inflammatory state of the joint, emerging single-cell metabolic profiling successfully unravels cellular heterogeneity by uncovering cell-type-specific lactate utilization features across distinct synovial subpopulations [134]. Furthermore, because hypoxic gradients exhibit a pronounced anatomical polarity within the joint, newly published spatial transcriptomics of the RA synovium provides a crucial spatial structural context, enabling us to map lactylation pathways directly onto specific localized cellular microenvironments [135]. Incorporating these cutting-edge multi-omics perspectives into this review establishes a spatially resolved, dynamic regulatory network, ultimately providing precise navigation for the design of future joint-targeted metabolic therapeutics.

## Data Availability

No new data were created or analyzed in this study.
